# Cyphenothrin (Veterinary Medicinal Products)

**DOI:** 10.14252/foodsafetyfscj.D-23-00008

**Published:** 2023-09-22

**Authors:** Food Safety Commission of Japan

## Abstract

Food Safety Commission of Japan (FSCJ) conducted a risk assessment of cyphenothrin (CAS No. 39515-40-7), a pyrethroid insecticide, intended to be used to exterminate cockroaches in piggeries. This was based on documents of pigsty sprays containing the active substance *d*∙*d*-T-Cyphenothrin submitted, and risk assessment reports of EPA (Environmental Protection Agency) and others. The data of *d*-T80-Cyphenothrin and *d∙d*-T-Cyphenothrin, with different abundance ratios of the eight optical isomers composing both cyphenothrins, were used for the evaluation. The data used in the assessment include pharmacokinetics (rats), residues (rats), genotoxicity, acute toxicity (mice and rats), subacute toxicity (mice, rats and dogs), chronic toxicity/carcinogenicity (mice, rats and dogs), reproductive toxicity (rats and rabbits), neurotoxicity (rats), general pharmacology and others. In the various genotoxicity tests, no genotoxicity of *d*-T80-Cyphenothrin were observed on living organisms. *d∙d*-T-Cyphenothrin was not expected to cause genotoxity from the results of *d*-T80-Cyphenothrin studies. FSCJ thus recognized it to be possible to specify an acceptable daily intake (ADI). The lowest no-observed-adverse-effect level (NOAEL) obtained from all the studies was 3 mg/kg bw per day. This value was based on the following effects of administration using *d*-T80-Cyphenothrin in dogs: Vomiting in a 13-week subacute toxicity study in males and females, and vomiting and redness of the oral mucous membranes in a 52-week chronic toxicity study in males. Addition of the safety factor 2 was appropriate based on the fact that the toxicity of *d∙d*-T-Cyphenothrin was slightly stronger than that of *d*-T80-Cyphenothrin. FSCJ thus specified an acceptable daily intake (ADI) of 0.015 mg/kg bw per day after applying a safety factor of 200 to the NOAEL.

## Conclusion in Brief

Food Safety Commission of Japan (FSCJ) conducted a risk assessment of cyphenothrin (CAS No. 39515-40-7), a pyrethroid insecticide, intended to be used to exterminate cockroaches in piggeries. This was based on documents of pigsty sprays containing the active substance *d∙d*-T-Cyphenothrin submitted, and risk assessment reports of EPA (Environmental Protection Agency) and others.

The data of *d*-T80-Cyphenothrin and *d∙d*-T-Cyphenothrin, with different abundance ratios of the eight optical isomers composing both cyphenothrins, were used for the evaluation. The data used in the assessment include pharmacokinetics (rats), residues (rats), genotoxicity, acute toxicity (mice and rats), subacute toxicity (mice, rats and dogs), chronic toxicity/carcinogenicity (mice, rats and dogs), reproductive toxicity (rats and rabbits), neurotoxicity (rats), general pharmacology and others.

In a pharmacokinetic study in rats, no differences were observed in the absorption, distribution and excretion between *d*-T80-Cyphenothrin and *d∙d*-T-Cyphenothrin. These results suggested the bioequivalence of both Cyphenothrins.

In the various genotoxicity tests, no genotoxicity of *d*-T80-Cyphenothrin were observed on living organisms. *d∙d*-T-Cyphenothrin was not expected to cause genotoxity from the results of *d*-T80-Cyphenothrin studies. FSCJ thus recognized it to be possible to specify an acceptable daily intake (ADI).

The neurological symptoms and suppression of body weight gain were observed as major adverse effects in subacute and chronic toxicity studies of *d*-T-80- and *d∙d*-T-Cyphenothrins.

Negative results were observed in carcinogenicity tests of *d*-T80-Cyphenothrin in mice and rats.

No teratogenicity was detected following the administration of *d*-T80-Cyphenothrin in developmental toxicity studies in rats and rabbits.

The lowest no-observed-adverse-effect level (NOAEL) obtained from all the studies was 3 mg/kg bw per day. This value was based on the following effects of administration using *d*-T80-Cyphenothrin in dogs: Vomiting in a 13-week subacute toxicity study in males and females, and vomiting and redness of the oral mucous membranes in a 52-week chronic toxicity study in males.

Addition of the safety factor 2 was appropriate based on the fact that the toxicity of *d∙d*-T-Cyphenothrin was slightly stronger than that of *d*-T80-Cyphenothrin.

FSCJ thus specified an acceptable daily intake (ADI) of 0.015 mg/kg bw per day after applying a safety factor of 200 to the NOAEL.
